# Application of the High-Throughput TAB-Array for the Discovery of Novel 5-Hydroxymethylcytosine Biomarkers in Pancreatic Ductal Adenocarcinoma

**DOI:** 10.3390/epigenomes3030016

**Published:** 2019-08-10

**Authors:** Chang Zeng, Zhou Zhang, Jun Wang, Brian C-H Chiu, Lifang Hou, Wei Zhang

**Affiliations:** 1 Department of Preventive Medicine, Northwestern University Feinberg School of Medicine, Chicago, IL 60611, USA; 2 Driskill Graduate Program in Life Sciences, Northwestern University Feinberg School of Medicine, Chicago, IL 60611, USA; 3 Department of Public Health Sciences, the University of Chicago, Chicago, IL 60637, USA; 4 The Robert H. Lurie Comprehensive Cancer Center, Northwestern University Feinberg School of Medicine, Chicago, IL 60611, USA

**Keywords:** pancreatic ductal adenocarcinoma, epigenetics, biomarker, 5-hydroxymethylcytosine

## Abstract

The clinical outcomes of pancreatic ductal adenocarcinoma (PDAC) remain dismal, with an estimated five-year survival rate of less than 5%. Early detection and prognostic approaches, including robust biomarkers for PDAC, are critical for improving patient survival. Our goal was to explore the biomarker potential of 5-hydroxymethylcytosines (5hmC), an emerging epigenetic marker with a distinct role in cancer pathobiology, yet under-investigated, due largely to technical constraints relating to PDAC. The TET-assisted bisulfite (TAB)-Array assay represents state-of-the-art technology and was used to directly profile 5hmC at single-base resolution with the Illumina EPIC array (~850,000 cytosine modification sites) in 17 pairs of tumor/adjacent tissue samples from US patients collected at the University of Chicago Medical Center. The TAB-Array data were analyzed to explore the genomic distribution of 5hmC and evaluate whether 5hmC markers were differentially modified between tumors and adjacent tissues. We demonstrated distinctive distribution patterns of 5hmC in tissue samples from PDAC patients relative to *cis*-regulatory elements (e.g., histone modification marks for enhancers), indicating their potential gene regulatory relevance. Substantial differences in 5hmC-modified CpG sites were detected between tumors and adjacent tissues in genes related to cancer pathobiology. The detected 5hmC-contaning marker genes also showed prognostic value for overall survival in the US patients with PDAC from the Cancer Genome Atlas Project. This study demonstrated the technical feasibility of the TAB-Array approach in cancer biomarker discovery and the biomarker potential of 5hmC for PDAC. Future studies using tissues and/or liquid biopsies may include 5hmC as a potential epigenetic biomarker target for PDAC.

## 1. Introduction

Pancreatic ductal adenocarcinoma (PDAC) ranks fourth in the United States and eighth worldwide for cancer mortality, with an estimated five-year survival rate of only 5%, in part because it is usually detected at the stage when it is no longer surgically resectable [1]. Reliable early detection and prevention is the only viable option for increased chances of survival but is currently unavailable for PDAC [2]. Due to the low incidence of PDAC in the population, most positive screening tests are likely false positives. In addition, the cancer biomarkers that are currently clinically available for PDAC, such as carbohydrate antigen (CA) 19-9, do not show satisfactory sensitivity and specificity for clinical utility [3]. Therefore, the identification of more effective biomarker targets for early detection, diagnosis, prognosis, and precise management of this fatal disease remains a great challenge.

Though the rapid advancement of high-throughput sequencing technologies has allowed for the characterization of tumor-related mutations, the low mutation frequency and lack of information on tissue of origin hampers detection sensitivity and specificity based on mutations alone. Recent studies have demonstrated aberrant cytosine modifications in tumors, suggesting potential applications of these epigenetic markers in cancer early detection and diagnosis [4,5]. In particular, 5-hydroxymethylcytosines (5hmC)—which are epigenetic marks that can be generated by oxidation of the more common 5-methylcytosines (5mC) through the TET (ten-eleven translocation) family of enzymes—have emerged as a stable type of modified cytosine in the human genome, covering ~0.1–1% of all CpG dinucleotides, with a distinct gene regulatory role and genomic distribution from 5mC [6,7,8]. The global reduction of 5hmC has been observed in both solid tumors and hematological malignancies, indicating its implications in cancer pathobiology [9,10,11,12,13]. Given their prevalence in the human genome, gene regulation relevance, and biochemical stability, the 5hmC markers have been exploited to be a novel class of epigenetic biomarkers for cancer diagnosis and prognosis [10,12,13,14,15]. 

Despite the remarkable potential of 5hmC as cancer biomarkers, their use in clinical samples has been limited mainly by the lack of enabling technologies that can distinguish 5hmC from other modified cytosines. For example, although the Illumina Infinium array has become a widely used epigenomic profiling platform, its conventional bisulfite-conversion protocol cannot distinguish 5hmC from 5mC. In the current study, we used the TET-assisted bisulfite (TAB)-Array [16], a state-of-the-art technique that is integrated with the Illumina EPIC array (covering ~850,000 CpG modification sites), to map and profile 5hmC modifications directly in a set of 17 paired tumor and adjacent tissue samples collected from US patients with primary PDAC [16,17]. This affordable yet high-throughput technique provided improved coverage of regulatory elements, thereby permitting more comprehensive detection of 5hmC-enriched regulatory elements [16]. Our aim was to demonstrate the technical feasibility of the TAB-Array in cancer biomarker discovery and the potential of 5hmC as novel epigenetic biomarkers for PDAC, thus laying the foundation for future development of clinically convenient assays for early detection of PDAC, for example, using emerging technologies in liquid biopsies [13,18]. 

## 2. Results

### 2.1. Genomic Distributions of 5hmC

**Figure 1:**
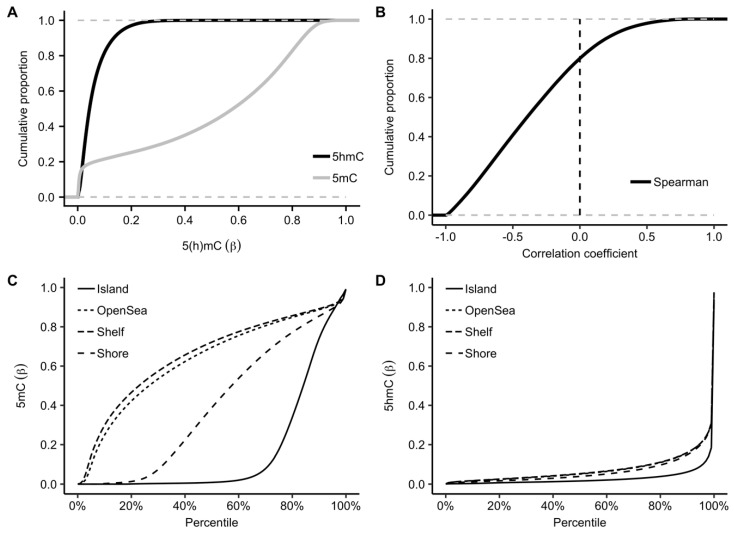
Profiled 5hmC loci show a distinct genomic pattern. In contrast to 5mC, 5hmC loci are, in general, biased toward the lower end of modification levels and show a distinct genomic distribution pattern of being enriched in gene bodies. (**A**) Comparison of the cumulative proportions between 5hmC and 5mC in terms of modification level (β-value); (**B**) 5hmC modifications are, in general, negatively correlated with 5mC; In (**A**,**B**), the dotted lines indicate boundaries of the cumulative proportion. Comparison of the genomic distributions between (**C**) 5mC and (**D**) 5hmC in PDAC tumors and adjacent tissues.

Using the TAB-Array assay, we profiled 5hmC modification levels in 17 pairs of tumor and adjacent tissue samples. Consistent with previous epigenetic studies using the Infinium arrays [14,16], the detected 5mC in PDAC tissues showed a two-mode distribution with peaks at both the lower and upper end of modification levels (<0.2 and >0.8 in terms of β-value). By contrast, the 5hmC modification sites showed a unimodal distribution enriched at the lower end of modification levels (Figure 1A). Notably, the 5hmC and 5mC levels were inversely correlated for the majority of the CpGs, while only ~20% of the CpGs demonstrated weak positive correlations (Figure 1B), indicating 5hmC has distinct roles from 5mC in tissue biology. Further separation of the modification sites into various genomic features showed enrichment of high 5mC modification levels within CpG islands (Figure 1C), in contrast to the enrichment of 5hmC observed in gene bodies, indicating distinct genomic distributions for these two types of cytosine modifications (Figure 1D).

### 2.2. PDAC-Associated 5hmC Loci

**Figure 2:**
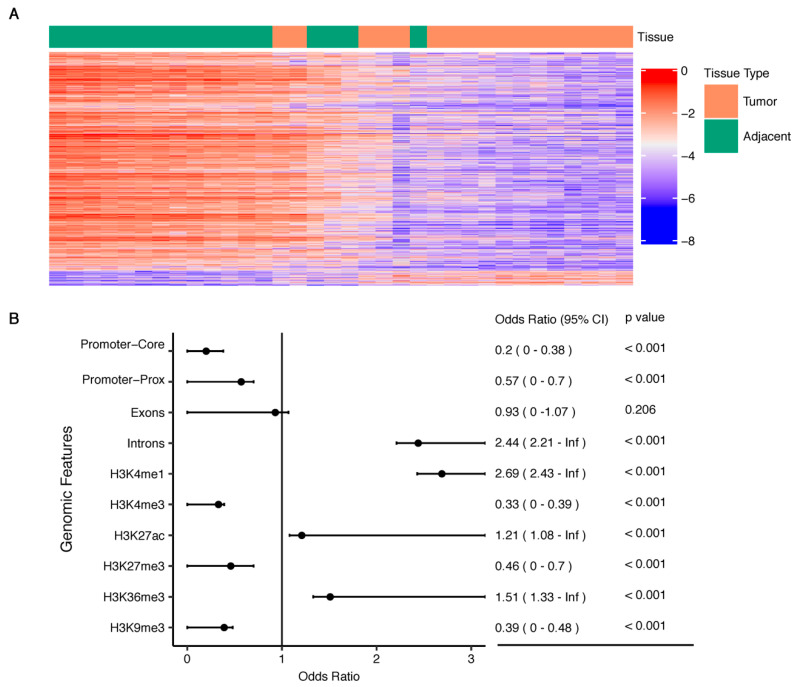
PDAC-associated 5hmC loci are enriched in gene bodies. (**A**) The heatmap of M-values ($M=\log_{2}(\frac{\beta }{1-\beta }$)) shows the detected 1118 differentially modified 5hmC loci (Bonferroni-corrected *p* < 0.05) between tumors and adjacent tissues; (**B**) The detected PDAC-associated 5hmC loci are enriched with gene bodies and histone modification marks for enhancers and active gene expression (e.g., H3K4me1, H3K27ac) derived from the pancreas from the Roadmap Epigenomics Project; Inf: infinity.

We identified 1118 differentially modified 5hmC loci among the 17 pairs of tumor and adjacent tissue samples with Bonferroni-corrected *p* < 0.05 (Figure 2A, Table S1). Examining the genomic distribution patterns of these PDAC-associated 5hmC loci suggested an overrepresentation in the genic regions, particularly gene bodies (Figure 2B). Enrichment analysis showed that the differentially modified 5hmC CpGs were more likely to reside in intronic regions while less likely to reside in promoter regions and exons. Based on the pancreas-derived data from the Roadmap Epigenomics Project [19], we further demonstrated that the detected PDAC-associated 5hmC loci were enriched with histone modification marks for enhancers and active gene expression regulation (Figure 2B). Specifically, we observed significant enrichment in enhancers (marked by H3K4me1), regions with active transcription (marked by H3K27ac), gene bodies (marked by H3K36me3), but depletion in promoters (marked by H3K4me3), regions with Polycomb repression (H3K27me3), and formation of heterochromatin (H3K9me3) (Figure 2B).

### 2.3. Exploring Functional Relevance, Prognostic Value, and Cancer Specificity

**Figure 3:**
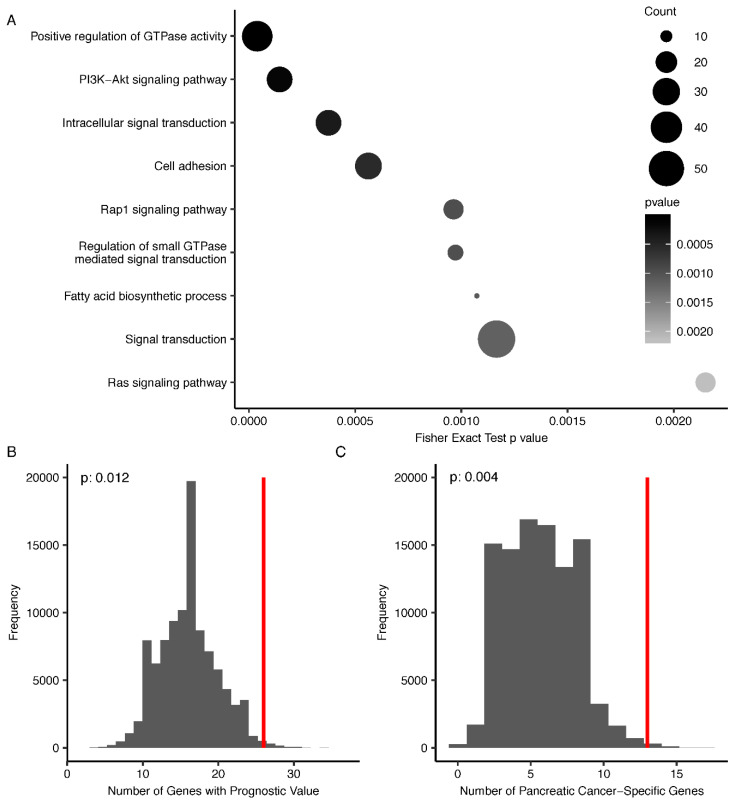
Functional relevance, prognostic value, and cancer specificity of PDAC-associated 5hmC loci. (**A**) The host genes containing PDAC-associated 5hmC loci are enriched in certain KEGG pathways and GO biological processes relevant to cancer pathobiology (Fisher’s exact test *p* < 0.01, gene count > 5); (**B**) Evaluation of prognostic value of PDAC-associated 5hmC loci using a permutation test (*n* = 100,000), based on the PDAC dataset from the Human Pathology Atlas (HPA) database. The real data point representing the number of PDAC-associated 5hmC loci with survival relevance in the HPA is shown as a red vertical line; (**C**) Evaluation of pancreatic cancer-specific gene expression of PDAC-associated 5hmC loci using a permutation test (*n* = 100,000) based on the PDAC dataset from the HPA database. The real data point representing the number of PDAC-associated 5hmC loci with elevated mRNA expression in PDAC in the HPA is shown as a red vertical line.

We searched the Kyoto Encyclopedia of Genes and Genomes (KEGG) database and Gene Ontology (GO) to explore functional relevance of the detected PDAC-associated 5hmC loci using the NIH/DAVID tool [20,21,22]. We found that those genes containing differentially modified 5hmC loci were enriched in several canonical pathways relevant to cancer pathobiology (Table S2) (*p* < 0.1, gene count > 5), such as PI3K-Akt signaling pathway (e.g., *FGF9*, *PDGFA*, and *TCL1B*), Rap1 signaling pathway (e.g., *MRAS*, *IGF1R*, and *ADCY9*), and Ras signaling pathway (e.g., *KSR2*, *EGF*, and *CALM2*); as well as GO biological processes, such as positive regulation of GTPase activity (e.g., *SMAP2, GIT2*, and *RSU1*) and fatty acid biosynthetic process (e.g., *PRKAG1*, *ELOVL7*, and *SCD5*) (Figure 3A) [23,24,25]. Specifically, 1048 out of 1118 PDAC-associated 5hmC loci have lower 5hmC modification levels in tumor tissues, and genes containing these differentially modified 5hmC loci were enriched in PI3K-Akt signaling pathway, focal adhesion, and Rap1 signaling pathway. By contrast, genes colocalized with 5hmC loci that have higher modification levels in tumors were enriched in cholinergic synapse (*GNAO1*, *GNG4*, and *GNG7*).

We further explored the potential prognostic value and pancreatic cancer specificity of those genes containing differentially modified 5hmC sites between PDAC tumors and adjacent tissues utilizing the transcriptomic data of 176 PDAC patients from the Cancer Genome Atlas (TCGA), as maintained by the HPA database [26,27]. Based on the five-year survival data, a total of 50 differentially modified 5hmC loci were located in those genes associated with patient survival (log-rank *p* < 0.001) in the HPA database (Table S1). A permutation test (*n* = 100,000) of the 822,760 CpGs sites interrogated by the EPIC array as background suggested that CpG sites with lower 5hmC modification levels in tumor samples were enriched in genes with “unfavorable” prognostic values for the TCGA PDAC samples (empirical *p* = 0.012) (Figure 3B). Based on the pan-cancer analysis from the HPA database, 194 genes were reported to have elevated mRNA levels as compared to those in the other 16 cancer tissues [27]. A similar permutation test (*n* = 100,000) of the 822,760 CpGs sites suggested these PDAC-associated 5hmC loci were also enriched with these expression signatures (the abovementioned 194 genes) of the TCGA PDAC samples (empirical *p* = 0.004) (Figure 3C). 

## 3. Discussion

Despite great efforts to improve its clinical outcomes, PDAC is expected to become the second leading cause of cancer death in the United States by 2030 [1]. To date, clinically available biomarkers such as CA19-9, though useful, do not yet demonstrate satisfactory sensitivity and specificity. Effective biomarkers, therefore, are urgently needed for PDAC early detection, diagnosis, prognosis, and disease surveillance to improve survival. Epigenetic regulators, including cytosine modifications, are particularly promising biomarkers for early detection of PDAC considering their tissue specificity, quantitative nature, and the availability of high-throughput profiling technologies. In the current report, using the recently developed innovative technique TAB-Array, we directly profiled 5hmC—the currently understudied cytosine modification type with demonstrated cancer relevance—in paired tumor and adjacent tissue samples from patients with PDAC. Our data showed a distinct genome-wide distribution pattern and modification landscape for 5hmC (Figure 1), consistent with previous studies [28]. Notably, we found that the detected 5hmC are, in general, inversely correlated with 5mC modifications, consistent with their putatively different gene regulatory roles. Notably, most previous epigenetic studies have either focused on 5mC or interpreted all modified cytosines as 5mC, and our findings support the importance of distinguishing 5hmC from 5mC in future epigenetic biomarker discovery assays for PDAC. A more precise understanding of the epigenetics of PDAC and epigenetic biomarker discovery would not be possible without taking into consideration of the distinct distributions and roles of these two different types of cytosine modifications.

Specifically, by comparing the 5hmC profiles between tumor and adjacent tissue samples, we showed a substantial number of 5hmC loci that were differentially modified at a stringent cutoff (Bonferroni-corrected *p* < 0.05) (Figure 2). Consistent with their putative gene regulatory roles and tissue specificity, these differential 5hmC modifications were significantly overrepresented in pancreas-derived histone modification marks, particularly for enhancer markers and active gene expression such as H3K4me1 and H3K27ac (Figure 2B). Moreover, cancer-related pathways such as the Ras and PI3K-Akt signaling pathways were enriched in PDAC-associated 5hmC sites (Table S2). Further examination of the HPA database suggested the overrepresentation of genes with prognostic value for five-year survival or genes with elevated expression, specifically in pancreatic cancer, among our PDAC-associated marker genes (Figure 3B,C), including such genes as *HDAC4*, *Rab1*, and *FIP3*, which are known to be related to PDAC pathobiology and/or metastasis [29,30]. Taken together, our findings demonstrated the technical feasibility of the TAB-Array in cancer biomarker discovery and indicated biomarker potential for 5hmC in the diagnosis and prognosis of PDAC.

We acknowledged there were several limitations of the current study. Firstly, the sample size was limited. Future investigation using more samples will elucidate the relevance of 5hmC in PDAC pathobiology. Secondly, the microarray-based profiling, though cost-effective, restricted the genome-wide scan to those modification sites available on the array. Future technical advances in next-generation sequencing-based approaches will allow for a more comprehensive and unbiased genome-wide analysis of 5hmC in PDAC. Thirdly, in the current report, we used the leftover samples from surgical resection to explore the biomarker potential of 5hmC. Future studies on patient samples with detailed clinical and epidemiological data will allow for a more comprehensive analysis of the relationships between 5hmC and other clinical/epidemiological factors. Finally, future investigations will need to elucidate the relationships between 5hmC profiles and tumor subtypes and to evaluate whether the 5hmC biomarkers are consistent across all tumors with different molecular characteristics. 

In summary, in the current study of 17 pairs of PDAC tumor and adjacent tissue samples, we demonstrated the feasibility of using the TAB-Array assay to profile genome-wide 5hmC with improved coverage and identify 5hmC-enriched genomic elements in PDAC tissues. A substantial number of 5hmC loci were found to be differentially modified between tumors and adjacent tissues from PDAC patients. Though limited by sample size and the lack of more detailed clinical data, our findings showed the technical feasibility of the TAB-Array in cancer biomarker discovery and functional relevance of those genes containing PDAC-associated 5hmC, as well as potential prognostic value of 5hmC markers. Overall, with the aid of the emerging technologies such as the TAB-Array, analysis of 5hmC is warranted as it is an important epigenetic target that can be used in future PDAC studies to facilitate the urgent need of developing effective biomarkers to improve clinical outcomes. 

## 4. Materials and Methods

### 4.1. Study Subjects

De-identified, clinically annotated leftover tissue samples from 17 adult patients with primary PDAC diagnosed during 2007–2011 (females: *n* = 10, males: *n* = 7; European descents: *n* = 7, African descents: *n* = 1, Hispanic: *n* = 1, Unknown races: *n* = 8; age at the time of diagnosis (mean ± standard deviation): 62.5 ± 15.8 years) were obtained from the Human Tissue Resource Center (HTRC) of the University of Chicago Medical Center. Patients treated with chemotherapy, radiation therapy, or immunotherapy were excluded from this study. Diagnosis of primary PDAC was histologically confirmed. Tumor and adjacent tissue samples were collected using the laser microdissection and snap-frozen at −80 °C at the HTRC. The Institutional Review Board of Northwestern University Feinberg School of Medicine (STU00200464, 05/02/2015) approved this study.

### 4.2. DNA Isolation and the TAB-Array Assay

Genomic DNA (gDNA) was isolated using the DNeasy Blood & Tissue kit (Qiagen, Germany), purified, and quantified, followed by analysis using the TAB-Array assay at the University of Chicago Genomics Core Facility [16]. Briefly, ~500 ng gDNA prepared from snap-frozen tissues was split into two fractions. One fraction was bisulfite-converted and analyzed by hybridization to the Illumina EPIC array (San Diego, CA, USA), which covers ~850,000 cytosine modification sites across the entire human genome. The second fraction was first glucosylated to protect the 5hmC from oxidation by recombinant Tet1 enzyme using the 5hmC-TAB-Array Kit (WiseGene LLC, Chicago, IL, USA), followed by bisulfite treatment and hybridization to the EPIC array. Unmethylated cytosines were converted to thymidines through bisulfite treatment. In the absence of TAB (Tet-assisted bisulfite) treatment, both 5hmC and 5mC are protected and remain as cytosines. TAB treatment thus protects 5hmC, while 5mC is oxidized and converted by sodium bisulfite to thymidines. The raw and processed TAB-Array data have been deposited into the NCBI/Gene Expression Omnibus database (Accession Number: GSE118694).

### 4.3. Processing of the TAB-Array Data and Detection of Differential Loci

The raw TAB-Array and conventional EPIC array data were processed using the R Package *ENmix*, separately [31]. Briefly, after the QC process, 3266 (TAB-Array) and 25,088 (bisulfite conversion protocol) low-quality CpGs (i.e., detection *p* > 10^−6^ in more than 5% samples) were detected for each assay, respectively. The detected low-quality CpGs were combined and removed from both datasets. For each dataset, the modification signal intensities were modeled with a flexible exponential-normal mixture distribution and the background noise was modeled with a truncated normal distribution for correction. Quantile normalization of modification intensities was performed across all 34 samples (17 pairs of tumor and adjacent tissues), and the β-values for each CpG probe were then obtained. The final 5hmC and 5mC β-values were estimated using the maximum likelihood estimate (MLE) from the paired bisulfite conversion and TAB-treated samples. The CpGs on sex chromosome were removed, resulting in a final set of 822,760 CpGs for further statistical analyses. To detect differentially modified 5hmC loci across the 17 pairs of tumor and adjacent tissue samples, the R Package *Limma* was used to perform a paired *t*-test for each probe [32]. Bonferroni-corrected *p* < 0.05 was used as the cutoff for statistical significance. All the statistical tests were performed under the R Statistical Computing Environment (v 3.5.1) [33]. 

### 4.4. Evaluating Genomic Distributions of 5hmC and Functional Relevance

To characterize the distributions of tumor-associated 5hmC loci, their genomic coordinates were extracted and mapped to their respective host genes based on the current GENCODE annotations (hg19) [34]. We also mapped the tumor-associated 5hmC loci to known histone modification marks derived from pancreas using ChIP-seq from the Roadmap Epigenomics Project [19]. Specifically, we retrieved the start and end annotations of the gapped peaks (i.e., narrow contiguous regions of enrichment for histone ChIP-seq with Poisson *p* < 0.01). The 5hmC loci that overlapped with these gapped peaks were considered to be colocalized with histone modification marks. To measure the enrichment of tumor-associated 5hmC loci with various genomic features (e.g., histone modification marks), the one-sided Fisher’s exact test was performed to calculate the odds ratio and *p*-value using the analyzed set of EPIC array probes (i.e., 822,760 CpGs) as the background reference. 

In addition, the biological relevance of the 5hmC-containing genes was explored using the NIH/DAVID tool (v 6.8) for the KEGG pathways and GO biological processes [20,21,22].

### 4.5. Exploring Prognostic Value and Pancreatic Cancer-Specific Expression of PDAC-Associated 5hmC Loci

The HPA is a database that explores the prognostic role of protein-coding genes in 17 different cancers by transcriptomics and antibody-based profiling [26]. The HPA predicts the prognostic role of a particular gene for PDAC using TCGA data on 176 US patients with primary PDAC [26]. According to the HPA, the “favorable” (or “unfavorable”) gene was defined as having higher relative expression levels at the time of diagnosis and while corresponding to higher (or lower for “unfavorable”) five-year overall survival, and the log-rank *p* < 0.001 was used to infer potential prognostic value of the 5hmC-contatining marker genes. 

Additionally, permutation tests were carried out to explore whether the detected PDAC-associated 5hmC loci were enriched with genes showing prognostic value or specifically expressed in PDAC. Random sampling was performed from the analyzed set of EPIC array probes (i.e., 822,760 CpGs) as background for 100,000 times to generate the null distribution based on the size of 5hmC-containing genes to be tested. To determine whether the PDAC-associated 5hmC loci were enriched in genes with prognostic value, the proportion of the simulations in which the number of genes with prognostic value was larger than the observed number was counted as the empirical *p*-value. Similarly, to determine whether PDAC-associated 5hmC loci was enriched in genes with specific expression in pancreatic cancer, the proportion of the simulations in which the number of genes with elevated mRNA expression levels in pancreatic cancer was larger than the observed number was counted as the empirical *p*-value.

## Supplementary Materials

Supplementary materials can be found at https://www.mdpi.com/2075-4655/3/3/16/s1.

## References

[1] Siegel R.L., Miller K.D., Jemal A. (2018). Cancer statistics, 2018. CA Cancer J. Clin..

[2] Del Chiaro M., Segersvard R., Lohr M., Verbeke C. (2014). Early detection and prevention of pancreatic cancer: Is it really possible today?. World J. Gastroenterol..

[3] Ballehaninna U.K., Chamberlain R.S. (2012). The clinical utility of serum CA 19-9 in the diagnosis, prognosis and management of pancreatic adenocarcinoma: An evidence based appraisal. J. Gastrointest. Oncol..

[4] Li J., Wu X., Zhou Y., Lee M., Guo L., Han W., Mo W., Cao W.M., Sun D., Xie R. (2018). Decoding the dynamic DNA methylation and hydroxymethylation landscapes in endodermal lineage intermediates during pancreatic differentiation of hESC. Nucleic Acids Res..

[5] Lomberk G., Blum Y., Nicolle R., Nair A., Gaonkar K.S., Marisa L., Mathison A., Sun Z., Yan H., Elarouci N. (2018). Distinct epigenetic landscapes underlie the pathobiology of pancreatic cancer subtypes. Nat. Commun..

[6] Yu M., Hon G.C., Szulwach K.E., Song C.X., Zhang L., Kim A., Li X., Dai Q., Shen Y., Park B. (2012). Base-resolution analysis of 5-hydroxymethylcytosine in the mammalian genome. Cell.

[7] Sun Z., Terragni J., Borgaro J.G., Liu Y., Yu L., Guan S., Wang H., Sun D., Cheng X., Zhu Z. (2013). High-resolution enzymatic mapping of genomic 5-hydroxymethylcytosine in mouse embryonic stem cells. Cell Rep..

[8] Song C.X., Szulwach K.E., Fu Y., Dai Q., Yi C., Li X., Li Y., Chen C.H., Zhang W., Jian X. (2011). Selective chemical labeling reveals the genome-wide distribution of 5-hydroxymethylcytosine. Nat. Biotechnol..

[9] Thomson J.P., Meehan R.R. (2017). The application of genome-wide 5-hydroxymethylcytosine studies in cancer research. Epigenomics.

[10] Li W., Zhang X., Lu X., You L., Song Y., Luo Z., Zhang J., Nie J., Zheng W., Xu D. (2017). 5-hydroxymethylcytosine signatures in circulating cell-free DNA as diagnostic biomarkers for human cancers. Cell Res..

[11] Pfeifer G.P., Xiong W., Hahn M.A., Jin S.G. (2014). The role of 5-hydroxymethylcytosine in human cancer. Cell Tissue Res..

[12] Cai J., Chen L., Zhang Z., Zhang X., Lu X., Liu W., Shi G., Ge Y., Gao P., Yang Y. (2019). Genome-wide mapping of 5-hydroxymethylcytosines in circulating cell-free DNA as a non-invasive approach for early detection of hepatocellular carcinoma. Gut.

[13] Zeng C., Stroup E.K., Zhang Z., Chiu B.C.H., Zhang W. (2019). Towards precision medicine: advances in 5-hydroxymethylcytosine cancer biomarker discovery in liquid biopsy. Cancer Commun..

[14] Johnson K.C., Houseman E.A., King J.E., von Herrmann K.M., Fadul C.E., Christensen B.C. (2016). 5-Hydroxymethylcytosine localizes to enhancer elements and is associated with survival in glioblastoma patients. Nat. Commun..

[15] Song C.X., Yin S., Ma L., Wheeler A., Chen Y., Zhang Y., Liu B., Xiong J., Zhang W., Hu J. (2017). 5-Hydroxymethylcytosine signatures in cell-free DNA provide information about tumor types and stages. Cell Res..

[16] Nazor K.L., Boland M.J., Bibikova M., Klotzle B., Yu M., Glenn-Pratola V.L., Schell J.P., Coleman R.L., Cabral-da-Silva M.C., Schmidt U. (2014). Application of a low cost array-based technique - TAB-Array - for quantifying and mapping both 5mC and 5hmC at single base resolution in human pluripotent stem cells. Genomics.

[17] Yu M., Hon G.C., Szulwach K.E., Song C.X., Jin P., Ren B., He C. (2012). Tet-assisted bisulfite sequencing of 5-hydroxymethylcytosine. Nat. Protoc..

[18] Kisiel J.B., Raimondo M., Taylor W.R., Yab T.C., Mahoney D.W., Sun Z., Middha S., Baheti S., Zou H., Smyrk T.C. (2015). New DNA methylation markers for pancreatic cancer: discovery, tissue validation, and pilot testing in pancreatic juice. Clin. Cancer Res..

[19] Roadmap Epigenomics C., Kundaje A., Meuleman W., Ernst J., Bilenky M., Yen A., Heravi-Moussavi A., Kheradpour P., Zhang Z., Wang J. (2015). Integrative analysis of 111 reference human epigenomes. Nature.

[20] Kanehisa M., Goto S. (2000). KEGG: kyoto encyclopedia of genes and genomes. Nucleic Acids Res..

[21] Dennis G., Sherman B.T., Hosack D.A., Yang J., Gao W., Lane H.C., Lempicki R.A. (2003). DAVID: database for annotation, visualization, and integrated discovery. Genome Biol..

[22] Ashburner M., Ball C.A., Blake J.A., Botstein D., Butler H., Cherry J.M., Davis A.P., Dolinski K., Dwight S.S., Eppig J.T. (2000). Gene ontology: tool for the unification of biology. The Gene Ontology Consortium. Nat. Genet..

[23] Baer R., Cintas C., Therville N., Guillermet-Guibert J. (2015). Implication of PI3K/Akt pathway in pancreatic cancer: When PI3K isoforms matter?. Adv. Biol. Regul..

[24] Lorenz R., Aleksic T., Wagner M., Adler G., Weber C.K. (2008). The cAMP/Epac1/Rap1 pathway in pancreatic carcinoma. Pancreas.

[25] Murthy D., Attri K.S., Singh P.K. (2018). Phosphoinositide 3-kinase signaling pathway in pancreatic ductal adenocarcinoma progression, pathogenesis, and yherapeutics. Front. Physiol..

[26] Uhlen M., Zhang C., Lee S., Sjostedt E., Fagerberg L., Bidkhori G., Benfeitas R., Arif M., Liu Z., Edfors F. (2017). A pathology atlas of the human cancer transcriptome. Science.

[27] Chang K., Creighton C.J., Davis C., Donehower L., Drummond J., Wheeler D., Ally A., Balasundaram M., Birol I., The Cancer Genome Atlas Research Network (2013). The Cancer Genome Atlas Pan-Cancer analysis project. Nat. Genet..

[28] Bhattacharyya S., Pradhan K., Campbell N., Mazdo J., Vasantkumar A., Maqbool S., Bhagat T.D., Gupta S., Suzuki M., Yu Y. (2017). Altered hydroxymethylation is seen at regulatory regions in pancreatic cancer and regulates oncogenic pathways. Genome Res..

[29] Giaginis C., Damaskos C., Koutsounas I., Zizi-Serbetzoglou A., Tsoukalas N., Patsouris E., Kouraklis G., Theocharis S. (2015). Histone deacetylase (HDAC)-1, -2, -4 and -6 expression in human pancreatic adenocarcinoma: associations with clinicopathological parameters, tumor proliferative capacity and patients’ survival. BMC Gastroenterol..

[30] He Y., Ye M., Zhou L., Shan Y., Lu G., Zhou Y., Zhong J., Zheng J., Xue Z., Cai Z. (2017). High Rab11-FIP4 expression predicts poor prognosis and exhibits tumor promotion in pancreatic cancer. Int. J. Oncol..

[31] Xu Z., Niu L., Li L., Taylor J.A. (2016). ENmix: A novel background correction method for Illumina HumanMethylation450 BeadChip. Nucleic Acids Res..

[32] Ritchie M.E., Phipson B., Wu D., Hu Y., Law C.W., Shi W., Smyth G.K. (2015). limma powers differential expression analyses for RNA-sequencing and microarray studies. Nucleic Acids Res..

[33] (2013). R: A Language and Environment for Statistical Computing. R Foundation for Statistical Computing. Vienna, Austria: R Foundation for Statistical Computing. https://scholar.google.ca/citations?user=yvS1QUEAAAAJ&hl=en.

[34] Harrow J., Frankish A., Gonzalez J.M., Tapanari E., Diekhans M., Kokocinski F., Aken B.L., Barrell D., Zadissa A., Searle S. (2012). GENCODE: the reference human genome annotation for The ENCODE Project. Genome Res..

